# Time Series Classification with InceptionFCN

**DOI:** 10.3390/s22010157

**Published:** 2021-12-27

**Authors:** Saidrasul Usmankhujaev, Bunyodbek Ibrokhimov, Shokhrukh Baydadaev, Jangwoo Kwon

**Affiliations:** Department of Electronic and Computer Engineering, Inha University, Incheon 22212, Korea; u.s.saidrasul@inha.edu (S.U.); bunyod.ibrokhimov@gmail.com (B.I.); sh.baydadaev@inha.edu (S.B.)

**Keywords:** deep neural networks (DNN), inception, fully convolutional network (FCN), time-series classification (TSC), optimization

## Abstract

Deep neural networks (DNN) have proven to be efficient in computer vision and data classification with an increasing number of successful applications. Time series classification (TSC) has been one of the challenging problems in data mining in the last decade, and significant research has been proposed with various solutions, including algorithm-based approaches as well as machine and deep learning approaches. This paper focuses on combining the two well-known deep learning techniques, namely the Inception module and the Fully Convolutional Network. The proposed method proved to be more efficient than the previous state-of-the-art InceptionTime method. We tested our model on the univariate TSC benchmark (the UCR/UEA archive), which includes 85 time-series datasets, and proved that our network outperforms the InceptionTime in terms of the training time and overall accuracy on the UCR archive.

## 1. Introduction

Time series classification started to attract attention in the early 2000s, and, at that time, the proposed methods were based on traditional, algorithm-based approaches. However, applying deep learning techniques for time-series data has recently become trendy amongst researchers. With the growth of data, the requirement of processing that data is also increasing. Therefore, there has been high demand among data mining researchers to extract, analyze, and understand the time-series data. However, with the implementation of deep neural networks, the capability of classifying the data also significantly increased. The idea of deep learning was first introduced by Yann LeCun [1] in 1998, when the multi-level artificial neural network was developed to classify the handwritten digits, but it truly gained popularity after the introduction of AlexNet [2] for image classification. Since then, tremendous research has been conducted that has led to the creation of successful algorithms. Deep neural networks are sophisticated and capable of solving computer vision problems by handling multidimensional data, such as image spatial information, for classification or localization. Most of the success of deep learning in image recognition tasks has been attributed to the “depth” of the architectures. Time series data, however, are computationally simpler and require the computation of only sequenced data.

UCR open-source archive, the largest time-series (TS) data collection, is used as a benchmark by most of the researchers to test the model performance. The UCR archive currently contains 156 datasets that were initially collected and normalized [3]. The datasets consist of different data lengths and numbers of classes. As a good example of the different methodologies to classify the TS data, we can outline Hassan Ismail Fawaz et al. [4], where the authors implemented various deep learning modules, including multilayer perceptron (MLP), encoder, residual network (ResNet), fully convolutional network (FCN), and compared the results with each and every dataset in the UCR archive for both univariate and multivariate data. Another upgraded network was proposed by the same authors [5], where they first adapted the Inception module for time series classification (TSC) in which each module has the same architecture with different randomly initialized weight values. The main idea behind the Inception module is to apply several simultaneous filters of varying length on a continuous time-series input. We investigated the diversity of the best-related work and found out that there are still topics to improve. For this study, we can decrease the training time without an accuracy drop by applying a wider number of filters to the fundamental layers inside the Inception block and modifying the hyperparameters. Instead, we added the computational overhead to amplify the performance. Additionally, we implemented deeper FCN with varying convolutional filters and finetuned the network by adding pooling layers and included a dropout layer to decrease the likelihood of overfitting and become less sensitive to smaller fluctuations in the time-series data.

The key contributions are summarized as follows:

1. Inception block modification—we modified the existing Inception module by finetuning the parameters for the convolutional and max-pooling layers. We created narrower convolutional layers than the original Inception block by comprising more kernels per layer. These changes speed up the training due to the decrease in the number of parameters and FLOPs [6].
2. Aggregation—we combined the deeper FCN block with the Inception module to boost the classification performance. We sequentially trained the initial time-series features on Inception and on FCN modules, then we merged the output with adding layers at the end of the network. Although each contribution is easy to be implemented, we believe this is the first work including the combination of both methods.

The rest of the paper is organized as follows. Section 2 briefly describes the UCR archive data collection and discusses various related work in traditional and deep learning methods. We explain the proposed network architecture in Section 3. Section 4 demonstrates the experimental results and illustrates numerical comparisons. Section 5 discusses our findings and the exclusion of some related work. Finally, Section 6 concludes the paper and discusses the possible future work.

## 2. Related Work

### 2.1. UCR Archive

The UCR archive was first introduced in 2002, and, since then, it has been an important resource for data miners. The archive itself previously consisted of 46 classes before a significant update in 2015, when researchers increased the number of datasets to 85. Additionally, the data were normalized and denoised. The latest update was completed in 2018 [7], with an expansion from 85 to 128 datasets. Sequential data were annotated, and a specific number was assigned as a class label. The number of classes for datasets varies from 2 to 60. According to Bing H. et al. [8], the datasets can be categorized into the time-series data definitions in which:

**Definition** **1.** *A univariate time-series is an ordered set of real-valued variables, and only local properties are being used in a time-series, and subsequences are enclosed.* $T=[T_{1},T_{2}\ldots T_{n}]$ *, where n is a sequence length with only one dimension X.*

**Definition** **2.** *Multivariate time-series with larger dimensions, which consist of number ordered elements* $\left[T=T_{1},T_{2}\ldots T_{n}\right]$ *and* $T_{i}\in \;{\mathbb{R}}^{X}$ *, where X is number of dimensions with n number or ordered elements.*

**Definition** **3.** *Set of data pairs where dataset can be either univariate or multivariate.*

(1) $$D=\left\{\left(A_{1},B_{1}\right),\;\left(A_{2},\;B_{2}\right),\;\ldots ,\left(A_{n},B_{n}\right)\right\}\;\mathrm{and}\;A_{i},B_{i}\in \;{\mathbb{R}}^{X};$$

*where X ≥ 1, thus, the data can also be presented in sets.*

### 2.2. Machine-Learning-Based Classification Algorithms

There are diverse classification algorithms; however, we considered describing three main categories for TSC with details of specific algorithms. These categories are based on:

1. Distance (K-means with dynamic time warping);
2. Interval (TimeSeriesForest);
3. Dictionary (BOSS, cBOSS).

K-means clustering with dynamic time warping is a well-known unsupervised learning method that constructs clusters of data by splitting samples into *k* groups and minimizing the sum-of-squares in each cluster. K-means algorithms are not always effective because they use the Euclidean algorithm that often produces pessimistic similarity measures when it encounters distortion in the time axis [9]. Replacing that algorithm with dynamic time warping (DTW) is one of the ways to enhance the classification performance. DTW finds the optimal non-linear alignment between two time-series data. DTW between two time-series data can be formulated as following optimization problem:

(2) $$DTW\left(a,b\right)=min_{P\;}\sqrt{\sum_{\left(i,j\right)\in P}d{\left(a_{i},\;b_{j}\right)}^{2}},\;\mathrm{thus}\;P=\left[p_{0},p_{1},\;\ldots \;p_{K}\right],$$

where *a* and *b* are time-series data in the domain of *P*. Thanawin Rakthanmanon et al. [10] implemented DTW algorithm to classify earlier UCR archives in 2012.

TimeSeriesForest (TSF) is a supervised learning method that is an ensemble of decision trees. Houtao Deng et al. [11] created a tree-ensemble method that employs a combination of entropy gain and a distance measure. TSF randomly samples features at each tree node and has computational complexity linear in the length of time series and can be built using parallel computing techniques. TSF is computationally efficient and outperforms DTW.

Bag-of-SFA-Symbols (BOSS) [12] is another method for classification that combines the extraction of substructures with the tolerance to data and noise reduction for time-series. The novelty in this approach was the Symbolic Fourier Approximations (SFA), which performs approximation and quantization operations [12]. The BOSS classifier is based on 1-nearest-neighbour (1-NN) classification. It searches for the 1-NN within a set of samples by minimizing the distance between the query and the input data.

However, with the advancement of the deep learning approaches, the above-mentioned distance (DTW), interval (TSF), and dictionary (BOSS) methods are being infrequently used.

### 2.3. Deep-Learning-Based TSC

All the algorithms listed above require some feature engineering methods as a separate task before classification and imply the higher loss of some necessary information within the processing time. On the other hand, in DL algorithms, features are directly learned via network training. Convolutional neural networks (CNN) show promising results for more complex computations, such as image classification and object recognition. However, unlike image, time-series input is fed to the convolutional layer with one-dimensional filters that can extract continuous discriminant features. In this section, we describe the most widely used DL techniques, such as:

1. Multi-layer perceptron (MLP);
2. Fully convolutional network (FCN);
3. Residual network (ResNet);
4. Inception module added networks (InceptionTime).

#### 2.3.1. Multi-Layer Perceptron for Classification

The feedforward neural network consists of multiple layers of neurons (perceptron). It computes the weighted sum of the input values and uses an activation function (sigmoid, tanh, ReLU) to the result. MLP is a fully connected network in which the output is obtained by computing in a sequence of the activations of a perceptron. Zhiguang Wang et al. [13] used basic MLP containing three fully connected layers with 500 neurons in each layer. They used dropout for each layer to improve generalizability. ReLU activation function is used to prevent saturation of the gradient descent for a deeper network. According to them, each layer block satisfies the following condition:

(3) $$x=f_{dropout,p}\left(x\right);y=W\cdot x+b;h=ReLU\left(y\right);$$

#### 2.3.2. Fully Convolutional Networks for Classification

Convolutional networks are mostly applicable to the images or multivariate time-series as they capture temporal and spatial patterns through the filters and assign the importance value to those patterns. Generally, FCNs have convolutional, batch normalization, pooling, and fully connected layers. The convolution is defined by a set of filters that are fixed-size matrices. According to Hassan Ismail Fawaz et al., FCN shows competitive quality and efficiency for TSC. FCN outperforms all the known deep learning methods, including ResNet. FCN for TSC was first introduced by Zhiguang Wang et al. [13], where they composed the network of three convolutional blocks and performed three consecutive operations on each block, such as convolution, batch normalization, and ReLU activation function. The output of the last third block is averaged with global average polling for the reduction in several parameters over the whole-time dimension.

Baydadaev et al. [14] proposed to use FCN for the vestibulo–ocular reflex (VOR) custom dataset. VOR data are the fixed-length multivariate time-series data that give information about head and eye velocities. Basically, the classification of the VOR data is based on the output gain; however, the difficulty of the classification was due to frequent irrelevant data that are considered as noise or artifacts. In that work, we proposed a 1D impulse classification network that could classify signals with 95% accuracy. Generally, the ICN network takes an input with a fixed length for signal data and is dependent on the amount of time-series data, quality of features, and the network architecture.

#### 2.3.3. Residual Networks for Classification

**Figure 1:**
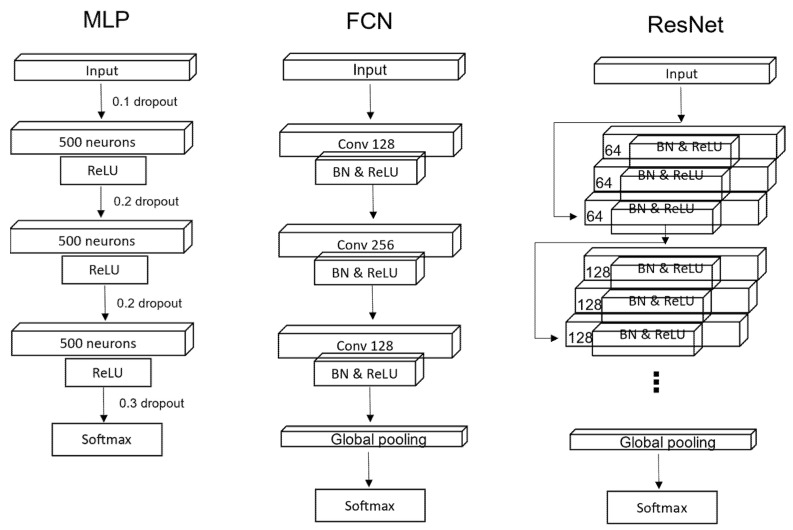
The generalization of network architectures of the different neural networks applied to classify time series data.

Residual networks are very deep structured networks that are easier to optimize. ResNet extends deeper by learning residual functions and having a shortcut connection to each residual block without learning all the unreferenced functions that enable the gradient flow directly through the bottom layers. ResNet for TSC was originally proposed by Wang et al. (2017) and was modified by Hassan Ismail Fawaz et al. As shown in Figure 1, the network architecture consists of three residual blocks, each with a convolutional layer, batch normalization layer and ReLu activation function, followed by a global average pooling layer with a softmax classifier at the bottom of the network. The number of convolutional filters in layers in the residual blocks have 64, 128, and 128 with the fixed filter length of 8, 5, and 3 for the first, second, and third blocks, respectively. ResNet has a constant number of parameters for various datasets.

#### 2.3.4. InceptionTime for Classification

Saeed Karimi-Bidhendi et al. mapped time-series data into Gramian angular difference field (GADF) images and used pretrained Inception v3 model to map those images into 2048-dimensional vector space. Then, they applied MLP to classify time-series data ([15]). Hassan Ismail Fawaz et al., however, proposed the state-of-the-art InceptionTime network that purely works on 1D time-series data. InceptionTime is a combination of five Inception networks, where they use the concept of the receptive field (RF), which is defined as the size of the region in the input that produces the feature. Most of the time, RF is used in CNN as a convolutional unit that depends on a local region of the input and is extensively used in the image segmentation [16]. Nevertheless, in our case, the input is 1D time-series data, and RF is computed by the formula:

(4) $$1+\overset{n}{\underset{i=1}{\sum }}\left(x_{i}-1\right),$$

where n is the network depth, and $x_{i}$ is filter length where i ∈[1,n]. The authors claimed that increasing the filter length for all the layers increases the RF for each network layer. Predictions made by a network with different initializations are computed by the formula:

(5) $$y_{i,c}=\frac{1}{n}\;\overset{n}{\underset{j=1}{\sum }}f_{c}\left(x_{i,\;\;}\theta_{j}\right)\;|\;\forall_{c}\;\in \left[1,\;c\right),$$

where $y_{i,c}$ denotes the output of the network for data $x_{i}$; which belong to a class c that can be either univariate or multivariate, and the function $f_{c}$ is averaged over *n* models.

## 3. Methodology

### 3.1. Network Architecture

**Figure 2:**
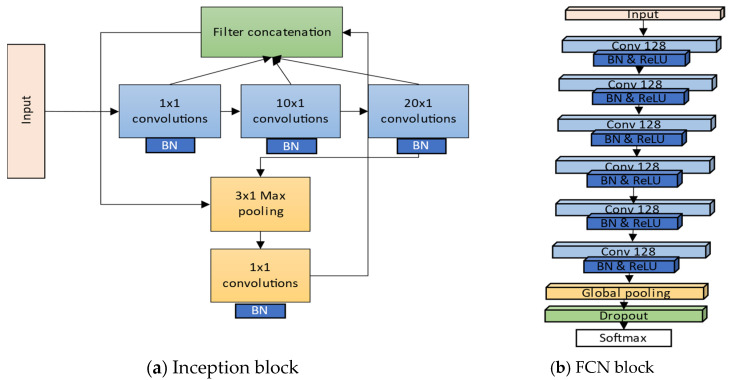
The overview of our Inception (**a**) and FCN (**b**) blocks.

Our proposed method has similarities with the InceptionTime method. The differences, however, include that we separated the network into two parts: the inception module and the shortcut module. The inception module is composed of two blocks. A bottleneck layer with a linear activation function is used at the top of the network to reduce the size of the input tensor in a convolutional layer that is a 1D convolutional layer with an input size of 64 and 1 × 1 kernel size. The rest of the block is similar to the original inception block and consists of the three convolutional layers with multiple filters of different sizes. Next, we added a max-pooling layer to make our model stable to small feature translation. Feature translation means that even if we reduce the dimensions of the feature map values, the output from the max-pooling layer does not change. Furthermore, another convolutional layer was added to extract hidden features from the sliding 1 × 1 filter. At the end of the block, there is a concatenating layer. We reduced the number of the inception blocks from three to two, and, unlike the InceptionTime network, our InceptionFCN has a much smaller number of trainable parameters as we applied convolutions of length {10, 20} instead of {40, 20, 10} as proposed. We avoided the use of the overparameterized network due to the risk of overfitting it. Fawaz et al. [5] created several models with architectural hyperparameters studies, where they modified the parameters of the inception blocks. In our proposed work, however, we chose optimal hyperparameters for the inception block to keep the training time and accuracy in the optimal trade-off. Instead, in the shortcut module, we applied deeper FCN, used in another study [14], with slight architectural changes. Implemented FCN consists of six 1D convolutional layers of the same size but with a different kernel length. The use of the deeper FCN compensates for the reduction of one inception block in the overall network performance. The initial data from the dataset are passed through the 1 × 1 kernel to every 128 filters on the first convolutional layer as an input. 1D convolutions can be performed to reach the desired number of labels. The loss of the FCN can be calculated by averaging the cross-entropy of every timestep and mini-batch. At the bottom of the shortcut block, we have the last layer in which we add the output tensor from the Inception module to the output tensor from the FCN module. Then, we perform global average pooling for the added tensor output, and, lastly, we perform a dropout to exclude the features from half of the nodes. The overview of the Inception module and FCN block are shown in Figure 2. Each convolutional layer is followed by batch normalization and ReLU activation function.

### 3.2. Training the Network with UCR Archive

Before training the network, the data from the UCR archive must be preprocessed due to the diversity in the number of classes, the data distribution, and intensity. We considered only the first definition data described in Section 2, which is the univariate time-series; therefore, we used a UCR archive consisting of 85 univariate datasets to fairly compare our experimental results with the existing methods. To adjust the data to the uniformly distributed one, we normalized the input data to the range of 0.0 to 0.1. We nullified the unavailable timestep values inside some datasets to maintain the integrity of the incoming data. Since the amount of the trainable data is very large, we used multithreading (parallelism) to fasten the preprocessing step. Our InceptionFCN network is scalable in the input layer as the input sizes are different in every dataset. InceptionFCN was trained with a single RTX2080 GPU computer; therefore, the training time may be different from that of Hassan Ismail Fawaz et al., where they used over 60 GPUs for training/testing. We used 1600 epochs for each class during training. Each dataset has a different training time since the input sizes and the number of classes is different. We included the early stopping method with the patience of 60 epochs. The median test accuracy was used as an evaluation metric. For comparison, we also trained the InceptionTime network on our machine with the hyperparameters presented by Hassan Ismail Fawaz et al. to check the processing time for both networks. We managed to decrease the training by reducing the number of trainable parameters. Using empirical trial-and-error mechanisms, the following hyperparameters are selected for training our network, as shown in Table 1.

## 4. Results

### 4.1. Evaluation Metric

We evaluated the overall accuracy, inference time, and FLOPs for the UCR-85 archive in the UCR benchmark. The number of characteristics (e.g., the number of classes, the size of the training/test, and time-series input size) vary according to each dataset. As an example, the authors of the UCR archive provided the per class error (PCE) for three classification types, such as 1-NN Euclidean distance, DTW, and 1-NN DTW, with no window wrapping as a benchmark. We calculated the PCE for each dataset to evaluate the classification metric on multiple datasets. PCE is found with the formula:

(6) $$PCE=\frac{1-acc}{n},$$

where *n* is the number of classes and *acc* refers to the classification accuracy.

### 4.2. Numerical Results and Comparison

To keep the comparison fair, we evaluated our network performance and selected the four best DL methods that claimed to be the SOTA results within recent years: InceptionTime, MLP, FCN, and ResNet. We have created the fairgrounds for each network and trained each network on our single GPU machine. Our network performed the best in both accuracy and performance tests, having the lowest PCE rate on the UCR-85 archive. From Table 2, we can see that our proposed network outperformed previous well-known methods by achieving higher accuracy for most of the datasets. In total, our model achieved results with Win/Tie/Loss of 52/9/17 out of 85 datasets, which is significant. InceptionTime also showed competitively good performance. However, our network is significantly faster in terms of training without affecting the overall performance of the network.

Table 3 shows the difference in the computational cost (FLOPs), which is proportional to inference and training time. Fewer total parameters than the InceptionTime provides a lower rate of overfitting the model, even on greater datasets. Our proposed method is trained significantly faster and has two times smaller architecture compared to the InceptionTime method (135 M vs. 309 M FLOPs) due to the fewer number of inception blocks and kernels.

### 4.3. Wilcoxon Signed-Ranks Test

We applied the Wilcoxon signed-ranks test, which is a nonparametric statistical hypothesis test. The test ranks the differences in the performances of two classifiers (ours versus InceptionTime) for each dataset and compares the ranks for the positive and negative differences (R+ and R−). This test helps evaluate the difference between the two methods. We set the null hypothesis as the two methods perform equally well for all 85 datasets, and the alternative hypothesis is that our model performs better than InceptionTime.

Let us assume $\partial_{i}$ to be the difference between the classifiers’ performance, where *I* is the classifier value on the *n*-th dataset. The differences are ranked from the lowest absolute value to the highest one. In the case of ties, average ranks are taken for those datasets. From this, we can calculate the *R*+ and *R*− by the following equations:

(7) $$R+=\overset{n}{\underset{\partial_{i}>0}{\sum }}rank\left(\partial_{i}\right)+\frac{1}{2}\overset{n}{\underset{\partial_{i}=0}{\sum }}rank\left(\partial_{i}\right)\;$$

(8) $$R-=\overset{n}{\underset{\partial_{i}<0}{\sum }}rank\left(\partial_{i}\right)+\frac{1}{2}\overset{n}{\underset{\partial_{i}=0}{\sum }}rank\left(\partial_{i}\right)\;$$

Let *T* be the smaller of the sums, *T* = *min* (*R*+, *R*−). General statistics include exact critical values for *T* for a large number of datasets, where:

(9) $$z=\frac{T-\frac{1}{4}n\left(n+1\right)}{\sqrt{\frac{1}{24}n\left(n+1\right)\left(2n+1\right)}}\;$$

Table 4 shows a performance comparison between InceptionFCN and InceptionTime. By calculating the difference, we can compute the ranks that are used to find the value of T. The sum of positive ranks is *R*+ = 954.5 (Equation (7)), and the sum of negative ranks is *R*− = 2710.5 (Equation (8)). This shows that our model outperforms InceptionTime because the difference is calculated by subtracting the InceptionTime accuracy from InceptionFCN accuracy. From *R*− and *R*+, we find that the value of *T* is 954.5. Note that the number of datasets used in this experiment is *n* = 85. Using Equation (9), we find that *z* is equal to −3.83, which is smaller than −1.96 for α= 0.05 using the *z*-score table. Therefore, we reject the null hypothesis and conclude that our method achieves better results compared to InceptionTime, as proposed.

The next step is to identify the statistical significance of this difference between the two methods. One way is to use a sign test (a form of the binomial test [17]) for wins and losses between the two best methods (since InceptionTime performs better than the other existing methods, we only compare the difference between InceptionTime and our InceptionFCN for this experiment). Under the null hypothesis, two compared methods should perform equally, which means each should win *n*/2 datasets. Since the number of wins is distributed according to a binomial distribution [18], we can use the z-test. For example, if one method wins at least in $\omega_{\alpha }$, shown in Equation (10), this method is considered significantly better than the other with *p* < 0.05.

(10) $$\omega_{\alpha }=\frac{1}{2}+z_{\alpha }*\;\sqrt{\frac{n}{2}}\;$$

for *n* = 85 and $z_{\alpha }$ = 1.96, the value of $\omega_{\alpha }$ is 51.54. From Table 3, it is seen that our method wins 55 (head-to-head comparison) datasets out of 85, which not only supports the alternate hypothesis (that our method outperforms InceptionTime) but also shows that the difference between these two methods is statistically significant with *p* < 0.05.

### 4.4. Critical Difference Calculation for Multiple Classifiers

**Figure 3:**
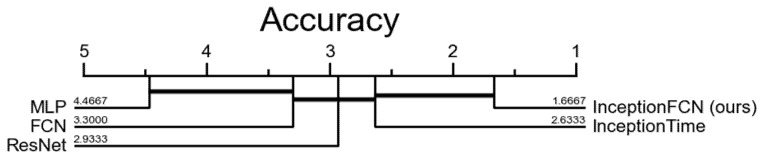
Critical difference diagram of the arithmetic means of the ranks for the selected DL methods.

Figure 3 shows the critical difference diagram for the compared methods.

For the multiple classifier comparisons, we used the Wilcoxon–Holm post hoc test to determine which classifiers are significantly different from one another. The average arithmetic rank represented in Figure 3 shows that our InceptionFCN is surpassing the well-known DL models. The critical difference diagram with Wilcoxon–Holm post-hoc analysis for the data presents the proof that adding deeper FCN to finetuned inception blocks improves the overall accuracy for the UCR archive. The critical difference is found by the following formula [19]:

(11) $$CD=q_{\alpha }\sqrt{\frac{y\left(y+1\right)}{6n}}\;$$

where *k* = 5 is the number of selected classifiers, $q_{\alpha }$ is the critical values that are based on the Studentized range statistic divided by $\sqrt{2}$, and *n* is the number of datasets.

## 5. Discussion

There are other DL methods that we did not consider for this research, such as LSTM-FCN by Fazle Karim et al. [20]. Their results outperformed most of the existing methods, and this approach is regarded as one of the first choices to address the DL-based time-series classification problem. However, the computational complexity is very high, particularly when many subsequent layers are used as LSTM networks can learn long-term relationships that go through geometric feature evolutions [21]. Therefore, a vast complexity increase was unacceptable for our research. For similar reasons, we did not consider implementing ResNet architecture. ResNet would be redundant when it is used with the Inception block since both blocks perform similarly. Moreover, Fawaz et al. already compared the performance of the InceptionTime with the ResNet model. Furthermore, our motivation for this research was to make a faster inferencing network with a time–accuracy trade-off.

Through various experiments, we showed that our proposed model could achieve a competitive performance while maintaining a smaller and more optimized network architecture. We believe this research will pave the way for many further research works directed at optimizing the network structure so that these methods can be implemented on small embedded devices with limited computational and memory resources.

## 6. Conclusions

In this paper, we enhanced a deep neural classifier for numerous time series classification tasks. Inspired by the Inception-based research, we evolved the inception module to achieve high performance and low computational cost (fewer FLOPs). We finetuned the network parameters and added a deeper shortcut FCN block to improve the performance for the TSC. Our approach is proven to be highly scalable as it can be applied to various time series collections of different sizes (i.e., UCR archive). The proposed method also simplified the network training as we reduced twice the number of parameters and conducted the current research using a single GPU machine. Moreover, using the Wilcoxon signed rank test and Wilcoxon–Holm post-hoc analysis, we showed that the InceptionFCN model outperforms InceptionTime significantly.

However, all the experiments were focused on the univariate datasets. For future work, we would like to expand our network to perform on multivariate data archives, such as UCR-128 and UCR-156. Moreover, we look forward to applying our architectural advancements in deep neural networks for various computer vision tasks.

## Figures and Tables

**Table 1:** Hyperparameters for InceptionFCN.

Entity	Value
Batch size	64
Bottleneck tensor size	64
Number of inception blocks	3
Number of epochs	1600
Kernel sizes for Inception block	[10, 20]
Kernel sizes for FCN block	[1,3,3,5,5,1]
Learning rate	$1e^{-3}$
Optimizer	Adam
Dropout rate	0.5

**Table 2:** Testing accuracy and per class error rate (PCE) for five different networks on UCR-85 dataset. Green color shows the winner in accuracy test.

Dataset	Ours	InceptionTime	MLP	FCN	ResNet
50words	0.771 (0.005)	0.829 (0.003)	0.684 (0.006)	0.627 (0.007)	0.74 (0.005)
Adiac	0.816 (0.005)	0.821 (0.005)	0.397 (0.016)	0.844 (0.004)	0.829 (0.005)
ArrowHead	0.88 (0.04)	0.829 (0.057)	0.778 (0.074)	0.843 (0.052)	0.845 (0.052)
Beef	0.767 (0.047)	0.7 (0.06)	0.72 (0.056)	0.697 (0.061)	0.753 (0.049)
BeetleFly	0.9 (0.05)	0.85 (0.075)	0.87 (0.065)	0.86 (0.07)	0.85 (0.075)
BirdChicken	1 (0)	1 (0)	0.775 (0.113)	0.955 (0.023)	0.885 (0.058)
Car	0.902 (0.025)	0.883 (0.029)	0.767 (0.058)	0.905 (0.024)	0.925 (0.019)
CBF	1 (0)	0.999 (0)	0.872 (0.043)	0.994 (0.002)	0.995 (0.002)
Chlorine Concentration	0.873 (0.042)	0.87 (0.043)	0.802 (0.066)	0.814 (0.062)	0.844 (0.052)
CinC_ECG_torso	0.857 (0.036)	0.854 (0.037)	0.84 (0.04)	0.824 (0.044)	0.826 (0.044)
Coffee	1 (0)	1 (0)	0.996 (0.002)	1 (0)	1 (0)
Computers	0.819 (0.091)	0.796 (0.102)	0.563 (0.219)	0.822 (0.089)	0.815 (0.093)
Cricket_X	0.835 (0.014)	0.826 (0.015)	0.591 (0.034)	0.792 (0.017)	0.791 (0.017)
Cricket_Y	0.853 (0.012)	0.851 (0.012)	0.6 (0.033)	0.787 (0.018)	0.803 (0.016)
Cricket_Z	0.834 (0.014)	0.851 (0.012)	0.617 (0.032)	0.811 (0.016)	0.812 (0.016)
DiatomSizeReduction	1 (0)	0.944 (0.014)	0.91 (0.023)	0.313 (0.172)	0.301 (0.175)
DistalPhalanxOutlineAgeGroup	0.81 (0.063)	0.795 (0.068)	0.657 (0.114)	0.71 (0.097)	0.717 (0.094)
DistalPhalanxOutlineCorrect	0.798 (0.101)	0.788 (0.106)	0.726 (0.137)	0.76 (0.12)	0.771 (0.115)
DistalPhalanxTW	0.765 (0.039)	0.74 (0.043)	0.617 (0.064)	0.69 (0.052)	0.665 (0.056)
Earthquakes	0.804 (0.098)	0.792 (0.104)	0.717 (0.142)	0.727 (0.137)	0.712 (0.144)
ECG200	0.94 (0.03)	0.93 (0.035)	0.916 (0.042)	0.889 (0.056)	0.874 (0.063)
ECG5000	0.938 (0.012)	0.941 (0.012)	0.929 (0.014)	0.94 (0.012)	0.934 (0.013)
ECGFiveDays	1 (0)	1 (0)	0.97 (0.015)	0.987 (0.007)	0.975 (0.013)
ElectricDevices	0.734 (0.038)	0.702 (0.043)	0.592 (0.058)	0.702 (0.043)	0.729 (0.039)
FaceAll	0.868 (0.009)	0.798 (0.014)	0.793 (0.015)	0.945 (0.004)	0.839 (0.012)
FaceFour	0.955 (0.011)	0.955 (0.011)	0.84 (0.04)	0.928 (0.018)	0.955 (0.011)
FacesUCR	0.956 (0.003)	0.962 (0.003)	0.833 (0.012)	0.946 (0.004)	0.955 (0.003)
FISH	0.994 (0.001)	0.983 (0.002)	0.848 (0.022)	0.958 (0.006)	0.979 (0.003)
FordA	0.94 (0.03)	0.949 (0.026)	0.73 (0.135)	0.904 (0.048)	0.92 (0.04)
FordB	0.936 (0.032)	0.932 (0.034)	0.603 (0.199)	0.878 (0.061)	0.913 (0.044)
Gun_Point	1 (0)	1 (0)	0.927 (0.037)	1 (0)	0.991 (0.005)
Ham	0.745 (0.128)	0.743 (0.129)	0.691 (0.155)	0.718 (0.141)	0.757 (0.122)
HandOutlines	0.964 (0.018)	0.893 (0.054)	0.918 (0.041)	0.806 (0.097)	0.911 (0.045)
Haptics	0.565 (0.087)	0.552 (0.09)	0.433 (0.113)	0.48 (0.104)	0.519 (0.096)
Herring	0.61 (0.195)	0.672 (0.164)	0.528 (0.236)	0.608 (0.196)	0.619 (0.191)
InlineSkate	0.502 (0.071)	0.453 (0.078)	0.337 (0.095)	0.339 (0.094)	0.373 (0.09)
InsectWingbeatSound	0.639 (0.033)	0.629 (0.034)	0.607 (0.036)	0.393 (0.055)	0.507 (0.045)
ItalyPowerDemand	0.969 (0.016)	0.965 (0.018)	0.954 (0.023)	0.961 (0.02)	0.963 (0.019)
LargeKitchenAppliances	0.901 (0.033)	0.893 (0.036)	0.473 (0.176)	0.902 (0.033)	0.9 (0.033)
Lighting2	0.742 (0.129)	0.836 (0.082)	0.67 (0.165)	0.739 (0.131)	0.77 (0.115)
Lighting7	0.863 (0.02)	0.822 (0.025)	0.63 (0.053)	0.827 (0.025)	0.845 (0.022)
MALLAT	0.964 (0.004)	0.963 (0.005)	0.918 (0.01)	0.967 (0.004)	0.972 (0.004)
Meat	0.952 (0.016)	0.933 (0.022)	0.897 (0.034)	0.853 (0.049)	0.968 (0.011)
MedicalImages	0.765 (0.024)	0.78 (0.022)	0.721 (0.028)	0.779 (0.022)	0.77 (0.023)
MiddlePhalanxOutlineAgeGroup	0.735 (0.088)	0.725 (0.092)	0.531 (0.156)	0.553 (0.149)	0.569 (0.144)
MiddlePhalanxOutlineCorrect	0.842 (0.079)	0.777 (0.112)	0.77 (0.115)	0.801 (0.1)	0.809 (0.096)
MiddlePhalanxTW	0.586 (0.069)	0.596 (0.067)	0.534 (0.078)	0.512 (0.081)	0.484 (0.086)
MoteStrain	0.943 (0.029)	0.895 (0.053)	0.858 (0.071)	0.937 (0.032)	0.928 (0.036)
NonInvasiveFatalECG_Thorax1	0.952 (0.001)	0.956 (0.001)	0.916 (0.002)	0.956 (0.001)	0.945 (0.001)
NonInvasiveFatalECG_Thorax2	0.951 (0.001)	0.961 (0.001)	0.917 (0.002)	0.953 (0.001)	0.946 (0.001)
OliveOil	0.85 (0.038)	0.7 (0.075)	0.667 (0.083)	0.723 (0.069)	0.83 (0.043)
OSULeaf	0.987 (0.002)	0.909 (0.015)	0.557 (0.074)	0.977 (0.004)	0.979 (0.004)
PhalangesOutlinesCorrect	0.824 (0.088)	0.846 (0.077)	0.735 (0.133)	0.82 (0.09)	0.839 (0.081)
Phoneme	0.341 (0.017)	0.322 (0.017)	0.096 (0.023)	0.325 (0.017)	0.334 (0.017)
Plane	1 (0)	1 (0)	0.978 (0.003)	1 (0)	1 (0)
ProximalPhalanxOutlineAgeGroup	0.851 (0.05)	0.834 (0.055)	0.856 (0.048)	0.831 (0.056)	0.853 (0.049)
ProximalPhalanxOutlineCorrect	0.921 (0.04)	0.921 (0.04)	0.733 (0.134)	0.903 (0.049)	0.921 (0.04)
ProximalPhalanxTW	0.802 (0.033)	0.77 (0.038)	0.767 (0.039)	0.767 (0.039)	0.78 (0.037)
RefrigerationDevices	0.552 (0.149)	0.52 (0.16)	0.379 (0.207)	0.508 (0.164)	0.525 (0.158)
ScreenType	0.61 (0.13)	0.581 (0.14)	0.403 (0.199)	0.625 (0.125)	0.622 (0.126)
ShapeletSim	0.994 (0.003)	0.956 (0.022)	0.503 (0.249)	0.724 (0.138)	0.779 (0.111)
ShapesAll	0.912 (0.001)	0.918 (0.001)	0.771 (0.004)	0.895 (0.002)	0.921 (0.001)
SmallKitchenAppliances	0.755 (0.082)	0.779 (0.074)	0.371 (0.21)	0.783 (0.072)	0.786 (0.071)
SonyAIBORobotSurface	0.929 (0.035)	0.887 (0.057)	0.672 (0.164)	0.96 (0.02)	0.958 (0.021)
SonyAIBORobotSurfaceII	0.987 (0.007)	0.962 (0.019)	0.834 (0.083)	0.979 (0.011)	0.978 (0.011)
StarLightCurves	0.978 (0.007)	0.977 (0.008)	0.949 (0.017)	0.961 (0.013)	0.972 (0.009)
Strawberry	0.972 (0.014)	0.966 (0.017)	0.961 (0.02)	0.972 (0.014)	0.981 (0.01)
SwedishLeaf	0.973 (0.002)	0.968 (0.002)	0.851 (0.01)	0.969 (0.002)	0.956 (0.003)
Symbols	0.985 (0.003)	0.977 (0.004)	0.832 (0.028)	0.955 (0.008)	0.906 (0.016)
synthetic_control	0.998 (0)	0.997 (0.001)	0.976 (0.004)	0.985 (0.003)	0.998 (0)
ToeSegmentation1	0.968 (0.016)	0.969 (0.016)	0.583 (0.209)	0.961 (0.02)	0.963 (0.019)
ToeSegmentation2	0.947 (0.026)	0.954 (0.023)	0.745 (0.128)	0.88 (0.06)	0.906 (0.047)
Trace	1 (0)	1 (0)	0.807 (0.048)	1 (0)	1 (0)
Two_Patterns	1 (0)	1 (0)	0.946 (0.014)	0.871 (0.032)	1 (0)
TwoLeadECG	1 (0)	0.996 (0.002)	0.762 (0.119)	1 (0)	1 (0)
uWaveGestureLibrary_X	0.811 (0.024)	0.815 (0.023)	0.767 (0.029)	0.754 (0.031)	0.78 (0.028)
uWaveGestureLibrary_Y	0.722 (0.035)	0.753 (0.031)	0.698 (0.038)	0.639 (0.045)	0.67 (0.041)
uWaveGestureLibrary_Z	0.752 (0.031)	0.759 (0.03)	0.697 (0.038)	0.726 (0.034)	0.75 (0.031)
UWaveGestureLibraryAll	0.887 (0.014)	0.941 (0.007)	0.955 (0.006)	0.817 (0.023)	0.86 (0.018)
wafer	1 (0)	0.999 (0.001)	0.996 (0.002)	0.997 (0.002)	0.999 (0.001)
Wine	0.744 (0.128)	0.611 (0.195)	0.565 (0.218)	0.587 (0.207)	0.744 (0.128)
WordsSynonyms	0.765 (0.009)	0.724 (0.011)	0.598 (0.016)	0.564 (0.017)	0.622 (0.015)
Worms	0.667 (0.067)	0.652 (0.07)	0.457 (0.109)	0.765 (0.047)	0.791 (0.042)
WormsTwoClass	0.748 (0.126)	0.713 (0.144)	0.601 (0.2)	0.726 (0.137)	0.747 (0.127)
yoga	0.898 (0.051)	0.896 (0.052)	0.855 (0.073)	0.839 (0.081)	0.87 (0.065)
Winner	52	26	2	12	15

**Table 3:** Average FLOPs comparison (in millions).

	InceptionFCN (Ours)	InceptionTime
Number of parameters (average)	198,432	423,222
FLOPs (average)	135 M	309 M

**Table 4:** Differences and ranks for two classifiers.

Dataset	Ours	InceptionTime	Difference	Rank
50words	0.771	0.829	0.058	76
Adiac	0.816	0.821	0.005	27.5
ArrowHead	0.88	0.829	−0.051	73
Beef	0.767	0.7	−0.067	79
BeetleFly	0.9	0.85	−0.05	72
BirdChicken	1	1	0	5
Car	0.902	0.883	−0.019	53
CBF	1	0.999	−0.001	15
ChlorineConcentration	0.873	0.87	−0.003	20
CinC_ECG_torso	0.857	0.854	−0.003	20
Coffee	1	1	0	5
Computers	0.819	0.796	−0.023	56
Cricket_X	0.835	0.826	−0.009	36.5
Cricket_Y	0.853	0.851	−0.002	17
Cricket_Z	0.834	0.851	0.017	50.5
DiatomSizeReduction	1	0.944	−0.056	75
DistalPhalanxOutlineAgeGroup	0.81	0.795	−0.015	48
DistalPhalanxOutlineCorrect	0.798	0.788	−0.01	40.5
DistalPhalanxTW	0.765	0.74	−0.025	58.5
Earthquakes	0.804	0.792	−0.012	45
ECG200	0.94	0.93	−0.01	40.5
ECG5000	0.938	0.941	0.003	20
ECGFiveDays	1	1	0	5
ElectricDevices	0.734	0.702	−0.032	63
FaceAll	0.868	0.798	−0.07	80
FaceFour	0.955	0.955	0	5
FacesUCR	0.956	0.962	0.006	30
FISH	0.994	0.983	−0.011	44
FordA	0.94	0.949	0.009	36.5
FordB	0.936	0.932	−0.004	24
Gun_Point	1	1	0	5
Ham	0.745	0.743	−0.002	17
HandOutlines	0.964	0.893	−0.071	81
Haptics	0.565	0.552	−0.013	46
Herring	0.61	0.672	0.062	77
InlineSkate	0.502	0.453	−0.049	71
InsectWingbeatSound	0.639	0.629	−0.01	40.5
ItalyPowerDemand	0.969	0.965	−0.004	24
LargeKitchenAppliances	0.901	0.893	−0.008	34.5
Lighting2	0.742	0.836	0.094	83
Lighting7	0.863	0.822	−0.041	67.5
MALLAT	0.964	0.963	−0.001	15
Meat	0.952	0.933	−0.019	53
MedicalImages	0.765	0.78	0.015	48
MiddlePhalanxOutlineAgeGroup	0.735	0.725	−0.01	40.5
MiddlePhalanxOutlineCorrect	0.842	0.777	−0.065	78
MiddlePhalanxTW	0.586	0.596	0.01	40.5
MoteStrain	0.943	0.895	−0.048	70
NonInvasiveFatalECG_Thorax1	0.952	0.956	0.004	24
NonInvasiveFatalECG_Thorax2	0.951	0.961	0.01	40.5
OliveOil	0.85	0.7	−0.15	85
OSULeaf	0.987	0.909	−0.078	82
PhalangesOutlinesCorrect	0.824	0.846	0.022	55
Phoneme	0.341	0.322	−0.019	53
Plane	1	1	0	5
ProximalPhalanxOutlineAgeGroup	0.851	0.834	−0.017	50.5
ProximalPhalanxOutlineCorrect	0.921	0.921	0	5
ProximalPhalanxTW	0.802	0.77	−0.032	63
RefrigerationDevices	0.552	0.52	−0.032	63
ScreenType	0.61	0.581	−0.029	60
ShapeletSim	0.994	0.956	−0.038	66
ShapesAll	0.912	0.918	0.006	30
SmallKitchenAppliances	0.755	0.779	0.024	57
SonyAIBORobotSurface	0.929	0.887	−0.042	69
SonyAIBORobotSurfaceII	0.987	0.962	−0.025	58.5
StarLightCurves	0.978	0.977	−0.001	15
Strawberry	0.972	0.966	−0.006	30
SwedishLeaf	0.973	0.968	−0.005	27.5
Symbols	0.985	0.977	-0.008	34.5
synthetic_control	0.998	0.997	−0.001	15
ToeSegmentation1	0.968	0.969	0.001	15
ToeSegmentation2	0.947	0.954	0.007	32.5
Trace	1	1	0	5
Two_Patterns	1	1	0	5
TwoLeadECG	1	0.996	−0.004	24
uWaveGestureLibrary_X	0.811	0.815	0.004	24
uWaveGestureLibrary_Y	0.722	0.753	0.031	61
uWaveGestureLibrary_Z	0.752	0.759	0.007	32.5
UWaveGestureLibraryAll	0.887	0.941	0.054	74
wafer	1	0.999	−0.001	15
Wine	0.744	0.611	−0.133	84
WordsSynonyms	0.765	0.724	−0.041	67.5
Worms	0.667	0.652	−0.015	48
WormsTwoClass	0.748	0.713	−0.035	65
yoga	0.898	0.896	−0.002	17

## Data Availability

Data available in a publicly accessible repository: the UCR time-series classification archive. Publicly available datasets were analyzed in this study. These data can be found at www.cs.ucr.edu/~eamonn/time_series_data/.
